# The association between serum uric acid and creatine phosphokinase in the general population: NHANES 2015–2018

**DOI:** 10.1186/s12872-023-03333-5

**Published:** 2023-06-12

**Authors:** Xinxin Chen, Jiuhong You, Mei Zhou, Hui Ma, Cheng Huang

**Affiliations:** 1 Department of Rehabilitation Medicine, West China Hospital, Sichuan University, Chengdu, Sichuan China; 2 Key Laboratory of Rehabilitation Medicine in Sichuan Province, West China Hospital, Sichuan University, Chengdu, Sichuan China; 3 School of Rehabilitation Sciences, West China School of Medicine, Sichuan University, Chengdu, Sichuan China; 4 Pesent Address: West China Hospital, Sichuan University, No. 37, Guoxue Alley, Wuhou District, Chengdu City, Sichuan Province China

**Keywords:** Uric acid, Creatine phosphokinase, NHANES, Cross-sectional study

## Abstract

**Background:**

The association between serum creatine phosphokinase (CPK), a standard biochemical measure of acute myocardial infarction, and serum uric acid (sUA) has not been studied. This study aimed to determine the association between sUA and CPK in the general population of the US.

**Methods:**

Data from the National Health and Nutrition Examination Survey (NHANES) 2015–2018 were used, including a total of 8,431 subjects aged ≥ 30 years. Weighted multiple regression analysis was used to estimate the independent relationship between sUA and CPK. Fitted smoothing curves and weighted generalized additive models were also performed.

**Results:**

We found a positive relationship between sUA and CPK after adjusting for potential confounders. In subgroup analyses stratified by sex and race/ethnicity, sUA was positively correlated with CPK in each subgroup. The association between sUA and CPK followed an inverted U-shaped curve in females (turning point: sUA = 428.3 μmol/L).

**Conclusions:**

Our study suggested that sUA level was positively correlated with CPK in the general population of the US. However, CPK increased with sUA until the turning point (sUA = 428.3 μmol/L) in females. Fundamental research and large sample prospective studies are needed to determine the exact mechanism of the association between sUA and CPK.

## Introduction

Creatine phosphokinase (CPK), also known as creatine kinase (CK), usually occurs in the heart, skeletal muscle, and brain [1]. It provides large amounts of ATP to cells and tissues [1]. Elevated CPK is a marker of free myoglobin following muscle cell injury and one of the oldest markers of acute myocardial infarction (AMI) [1, 2]. The CPK activity increases rapidly in conditions such as AMI and trauma to skeletal muscle [3]. Family history, physical examination, laboratory test results (especially CPK) and electrocardiogram results are often important ‘red flags’ for the diagnosis of cardiomyopathy [4]. Myocardial cell destruction, as in the setting of AMI, causes elevation of total CPK [5]. Currently, CPK remains the standard biochemical measure used in the diagnosis and treatment of AMI [6]. AMI is the most serious manifestation of coronary artery disease and the leading cause of morbidity and mortality worldwide, affecting more than seven million people worldwide each year [7]. Therefore, it is necessary to explore the relationship between AMI markers and other biochemical indicators.

Serum uric acid (sUA) is the final product of purine nucleotide metabolism in the human body, and hyperuricemia is caused by excessive synthesis or insufficient excretion of sUA [8]. The prevalence of hyperuricemia has increased in recent years, especially in high-income countries and economically developing countries with a Western lifestyle [9, 10]. According to a previous nationally representative survey, the prevalence of hyperuricemia in the United States was 20.2% in men and 20.0% in women between 2015 and 2016 and remained stable from 2007 to 2017 [11]. In addition to being a risk factor for gout, chronic inflammatory arthritis, metabolic syndrome, and kidney disease [12–14], studies have shown that elevated sUA is an independent risk factor for cardiovascular disease (CVD), including coronary artery disease and MI [15, 16]. Hyperuricemia promotes the onset and progression of CVD by modulating molecular signals such as the inflammatory response, oxidative stress, insulin resistance/diabetes, endoplasmic reticulum stress, and endothelial dysfunction [17]. The Rotterdam Study and the Apolipoprotein Mortality Risk Study (AMORIS) showed that high sUA levels were associated with a high risk of MI [18]. Mandurino-Mirizzi et al. found that elevated sUA may affect myocardial reperfusion in patients with MI and was associated with larger infarct size and higher long-term mortality [19]. Patients with hyperuricemia showed a larger infarct size and experienced a poorer prognosis after MI than non-hyperuricemia patients [20].

To the best of our knowledge, no one has previously explored the correlation between sUA and CPK. Therefore, we conducted this population-based cross-sectional study to investigate the association between sUA levels and CPK in a large US population.

## Materials and methods

### Study design

This was a cross-sectional study using the National Health and Nutrition Examination Survey (NHANES) (2015–2018), which is conducted every two years by the US Centers for Disease Control and Prevention. It is a nationwide population-based survey to assess the health and nutritional status of the US population. NHANES implemented a cross-sectional study design with complex multistage, stratified, and clustered sampling strategies. More details about the NHANES are available at www.cdc.gov/nchs/nhanes/.

### Study population

**Fig. 1:**
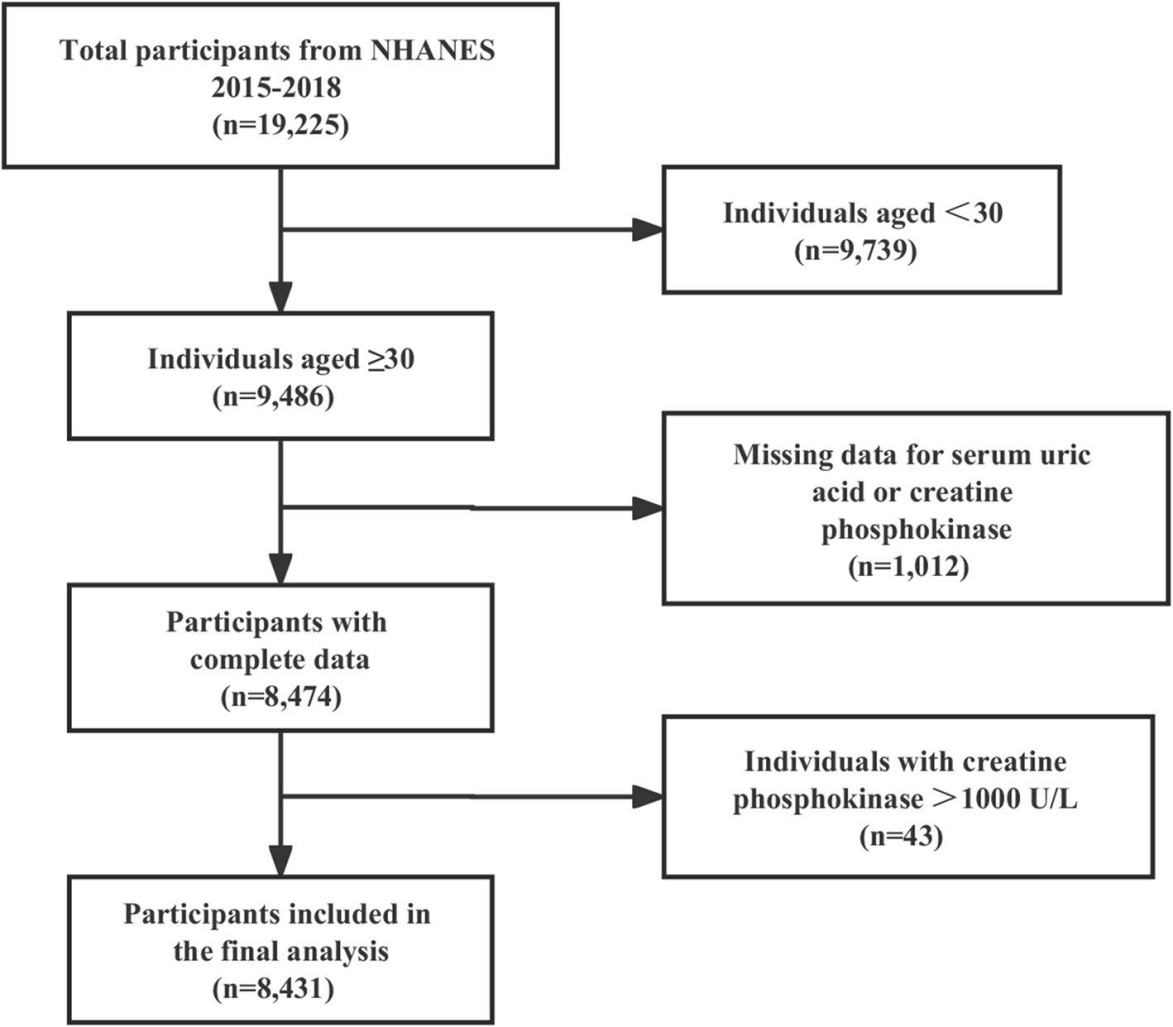
Sample screening flow chart

Nineteen thousand two hundred twenty-five participants were identified from NHANES 2015–2018. The study population was limited to participants aged ≥ 30 years with complete data on sUA and CPK. The exclusion criteria were those who have missing sUA data (*n* = 1,004) or CPK data (*n* = 8) and CPK > 1000 U/L (*n* = 43). Ultimately, 8,431 subjects remained for the final analysis (Fig. 1).

The NHANES study was approved by the National Center for Health Statistics (NCH) Ethics Review Board, and written informed consent was obtained from all participants.

### Variables

The main variables of this study were sUA and CPK, which were the independent variable and dependent variable, respectively. sUA and CPK were both items in the standard biochemical examination and were measured at the same time. In the NHANES 2015–2016 cycle, sUA was measured by the DxC800 using a timed endpoint method, and CPK was measured by the DxC800 using an enzymatic rate method. In the NHANES 2016–2018 cycle, sUA and CPK were measured on a Roche Cobas 6000 (c501 module) analyzer, and the methods were based on the same principles used in the 2015–2016 cycle.

Additionally, the following covariates were included: age, sex, race/ethnicity, education level, income-poverty rate, body mass index (BMI), waist circumference, physical activity, smoking and alcohol behavior, alanine aminotransferase (ALT), aspartate aminotransferase (AST), high-density lipoprotein cholesterol(HDL-C), low-density lipoprotein cholesterol(LDL-C), triglycerides (TG), total cholesterol (TC), total protein, hypertension, diabetes, and weak/failing kidneys. Data on age, sex, race/ethnicity, education level, and income-poverty rate were obtained from the demographic questionnaire. Race/ethnicity was classified as white, black, Mexican American, and other race/ethnicity. Education level was categorized as having completed less than high school, high school, and college graduate or above. Data on BMI and waist circumference were obtained from the physical examination section of NHANES. A BMI of < 25, ≥ 25, and ≥ 30 kg/m^2^ was defined as under/normal weight, overweight, and obesity, respectively. Waist circumference was measured at the navel using an elastic tape (to the closest 0.1 cm), with the participants standing at the end of a normal exhalation. Data on physical activity (sports, fitness, and recreation activities) were obtained from the physical activity questionnaire. According to the question ‘In a typical week do you do any moderate-intensity sports, fitness, or recreational activities that cause a small increase in breathing or heart rate such as brisk walking, bicycling, swimming, or volleyball for at least 10 min continuously’, the physical activity was categorized as yes and no. According to the question ‘Have you smoked at least 100 cigarettes in your life?’, the smoking status was divided into yes and no. Drinking status was classified as none or rarely (rarely or none), sometimes (1 to 3 times a month), and often (≥ 1 time a week). Hypertension was defined as mean systolic blood pressure ≥ 130 mmHg, mean diastolic blood pressure ≥ 80 mmHg, ongoing use of antihypertensive drugs, or self-reported doctor-diagnosed hypertension. Diabetes was defined as self-reported doctor-diagnosed diabetes or current use of insulin or diabetes medications. Weak/failing kidney disease was defined as self-reported physician-diagnosed weak/failing kidneys. It does not include kidney stones, bladder infections, or incontinence.

### Statistical analysis

All statistical analyses were performed using EmpowerStats (X&Y Solutions, Boston, MA) and R (version 3.4.3). The NHANES sample weights were taken into account when calculating the estimates. Categorical variables are presented as frequencies or percentages, and continuous variables are reported as the mean ± standard deviation. sUA was shown as a continuous variable, and divided into quartiles.

After adjusting for potential confounders, weighted multiple regression analysis was used to estimate the independent relationship between sUA and CPK. Then, the nonlinear relationship between sUA and CPK in the subgroup analyses was described by weighted generalized additive models and smooth curve fitting. Tests for interaction were performed with likelihood ratio tests. Two-piece linear regression models were further applied to examine the threshold effect of sUA on CPK after adjusting for all covariates. Weighted linear regression models and weighted chi-square tests were used to calculate differences between different groups for continuous variables and categorical variables, respectively. *p* < 0.05 was considered statistically significant.

## Results

### Characteristics of participants

A total of 8,431 adults aged ≥ 30 years were selected from the 2015–2018 NHANES for analysis in this study. The characteristics of the included subjects were subdivided into sUA quartiles. Of these subjects, 47.6% were males, 65.7% were Whites, 10.2% were Blacks, and 8.0% were Mexican Americans. Significant differences in age, sex, race/ethnicity, education level, BMI, waist circumference, physical activity, smoking and alcohol behavior, ALT, AST, CPK, HDL-C, LDL-C, TG, TC, total protein, hypertension, diabetes, and weak/failing kidneys were observed among the different groups of sUA (quartiles, Q1–Q4) (Table 1). sUA levels increased with age (*p* < 0.001). Waist circumference showed an increased linear trend in relation to sUA levels (*p* < 0.001). Serum ALT, AST, CPK, TG, and total protein increased linearly with the increasing sUA levels (*p* < 0.001 for all). People who were male, obese, had high blood pressure, had diabetes, or smoked/drank were more likely to have high sUA levels.

**Table 1:** Characteristics of participants

Serum uric acid (μmol/L)	Total	Q1	Q2	Q3	Q4	*p* Value
**Age (years)**	53.4 ± 14.3	51.3 ± 13.9	52.9 ± 14.2	54.4 ± 14.0	55.0 ± 14.8	< 0.001
**Gender (%)**						< 0.001
Male	47.6	15.9	39.7	60.1	75.2	
Female	52.4	84.1	60.3	39.9	24.8	
**Race/ethnicity (%)**						< 0.001
White	65.7	64.8	65.7	66.9	65.5	
Black	10.2	9.4	9.2	10.1	12.2	
Mexican American	8.0	9.8	8.1	7.8	6.5	
Other	16.0	16.0	17.0	15.2	15.8	
**Educational level (%)**						< 0.001
Less than high school	13.1	13.4	11.9	13.2	13.9	
High school	22.8	18.9	23.2	23.8	25.1	
College graduate or above	64.1	67.6	64.8	63.0	61.0	
**Income poverty ratio**	3.1 ± 1.6	3.1 ± 1.6	3.1 ± 1.5	3.2 ± 1.6	3.1 ± 1.6	0.231
**BMI (%)**						< 0.001
Normal: < 25 (kg/m^2^)	17.9	31.6	20.6	12.6	6.8	
Overweight: BMI ≥ 25 and < 30 (kg/m^2^)	25.4	28.1	26.1	25.5	21.6	
Obesity: BMI ≥ 30(kg/m^2^)	55.7	39.6	52.0	61.3	70.2	
**Waist circumference (cm)**	102.4 ± 16.0	94.6 ± 14.3	100.3 ± 14.9	105.0 ± 15.1	109.7 ± 15.9	< 0.001
**Physical activity (%)**						0.037
Yes	46.6	49.2	46.0	46.5	44.7	
No	53.4	50.8	54.0	53.4	55.3	
**Smoking behavior (%)**						< 0.001
Yes	45.0	40.3	42.9	47.2	50.0	
No	54.9	59.7	57.0	52.8	50.0	
**Alcohol behavior (%)**						< 0.001
None or rarely	25.3	28.6	26.3	24.1	22.3	
Sometimes	18.6	19.6	16.7	18.8	19.4	
Often	29.3	22.2	28.0	32.3	35.0	
Missing data	26.7	29.7	29.0	24.8	23.2	
**AST(U/L)**	23.9 ± 13.3	21.8 ± 9.8	22.9 ± 15.9	24.2 ± 11.5	26.7 ± 14.2	< 0.001
**ALT (U/L)**	24.1 ± 15.8	19.9 ± 11.9	22.1 ± 14.2	25.4 ± 15.7	29.4 ± 18.9	< 0.001
**CPK (IU/L)**	139.0 ± 120.7	108.3 ± 86.1	127.7 ± 99.9	147.6 ± 121.0	173.5 ± 156.2	< 0.001
**HDL-C (mmol/L)**	1.4 ± 0.5	1.6 ± 0.5	1.5 ± 0.5	1.3 ± 0.4	1.3 ± 0.4	< 0.001
**TC (mmol/L)**	5.0 ± 1.1	5.0 ± 1.0	5.1 ± 1.1	5.0 ± 1.0	5.1 ± 1.1	0.049
**TG (mmol/L)**	1.3 ± 0.7	1.2 ± 0.6	1.3 ± 0.6	1.4 ± 0.6	1.5 ± 0.8	< 0.001
**LDL-C (mmol/L)**	2.9 ± 0.6	2.9 ± 0.6	2.9 ± 0.6	3.0 ± 0.7	3.0 ± 0.7	0.011
**Total protein (g/L)**	70.8 ± 4.4	70.1 ± 4.3	70.6 ± 4.2	71.1 ± 4.3	71.4 ± 4.5	< 0.001
**Hypertension (%)**						< 0.001
Yes	38.0	29.5	33.3	38.0	51.8	
No	61.9	70.5	66.7	61.8	48.1	
**Diabetes (%)**						< 0.001
Yes	13.6	11.4	13.4	11.4	18.3	
No	83.7	85.9	84.4	85.3	78.9	
**Weak/failing kidneys**						< 0.001
Yes	3.5	2.1	2.7	3.6	5.6	
No	96.4	97.3	97.3	96.3	94.1	

Mean ± SD for continuous variables: The *p* value was calculated by a weighted linear regression model. (%) for categorical variables: *p* value was calculated by weighted chi-square test

*Abbreviations*: *BMI* body mass index, *AST* aspartate aminotransferase, *ALT* alanine aminotransferase, *HDL-C* high-density lipoprotein cholesterol, *TC* total cholesterol, *TG* triglycerides, *LDL-C* low-density lipoprotein cholesterol

### Association between sUA and CPK

**Fig. 2:**
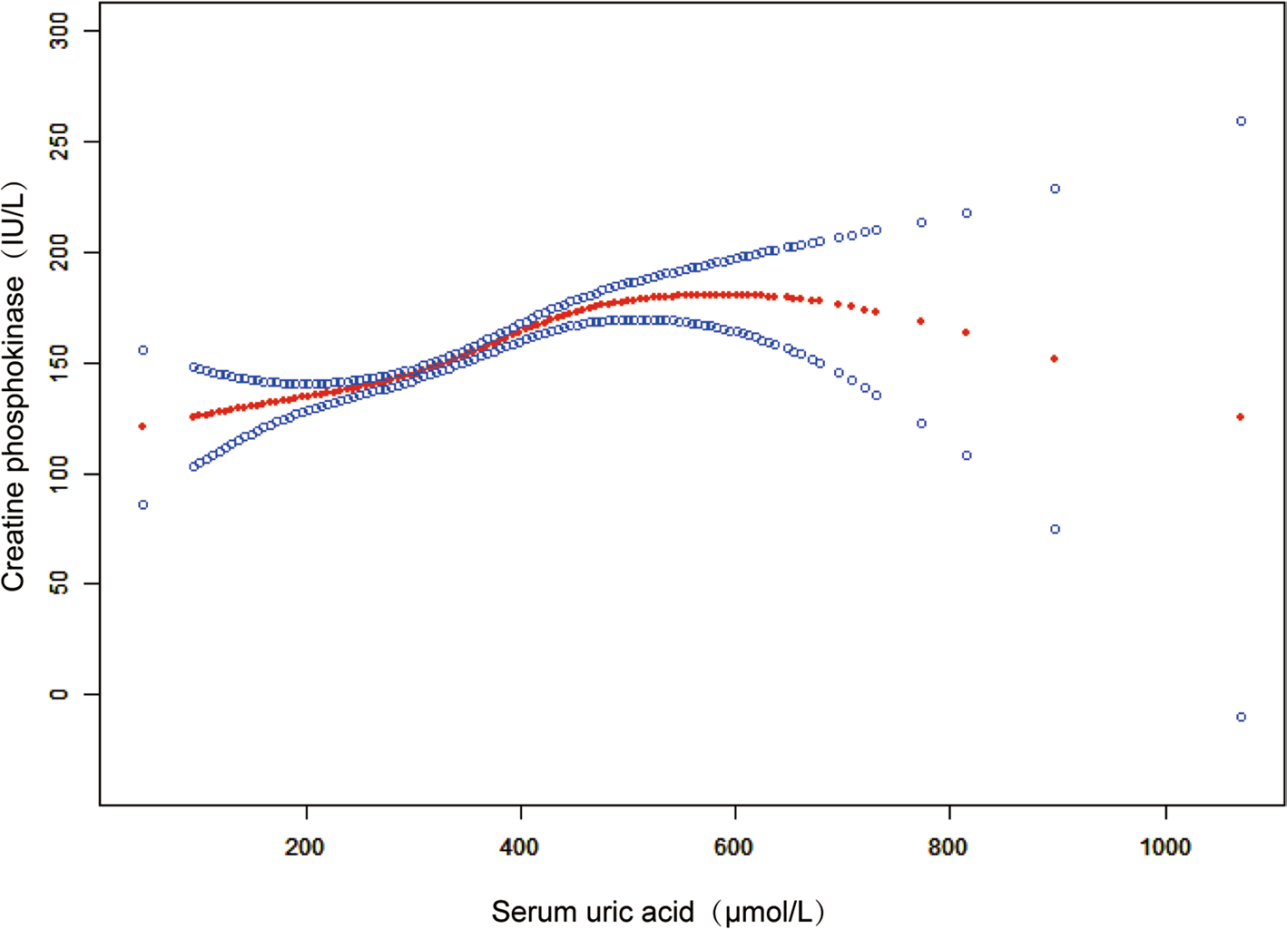
Association between serum uric acid and creatine phosphokinase. The area between two blue dotted lines is expressed as a 95% CI. Age, sex, race/ethnicity, education level, income poverty ratio, BMI, waist circumference, physical activity, smoking behavior, alcohol behavior, alanine aminotransferase, aspartate aminotransferase, high-density lipoprotein cholesterol, total cholesterol, triglycerides, low-density lipoprotein cholesterol, total protein, hypertension, diabetes, and weak/failing kidneys were adjusted. Significance of smooth terms: *p* < 0.001

The results of the multivariate regression analyses are shown in Table 2. Three models were established: model 1, unadjusted; model 2, sex, age, and race were adjusted; and model 3, adjusted for covariates, as shown in Table 1. In the fully adjusted model, sUA and CPK were positively associated [0.12(0.09, 0.15), *p* < 0.001] (Table 2, Fig. 2). When stratifying by gender or race/ethnicity, there was still a positive correlation between sUA and CPK.

**Table 2:** The association between serum uric acid (μmol/L) and creatine phosphokinase (IU/L)

	**Model 1** **β (95% CI), *p* value**	**Model 2** **β (95% CI), *p* value**	**Model 3** **β (95% CI), *p* value**
**Serum uric acid (μmol/L)**	0.30(0.27, 0.33), < 0.001	0.14 (0.11, 0.17), < 0.001	0.12 (0.09, 0.15), < 0.001
**Subgroup analysis stratified by gender**			
Male	0.23 (0.17, 0.28), < 0.001	0.20 (0.15, 0.26), < 0.001	0.15 (0.10, 0.21), < 0.001
Female	0.07 (0.04, 0.10), < 0.001	0.05 (0.02, 0.08), 0.002	0.05 (0.01, 0.08), 0.009
**Subgroup analysis stratified by race/ethnicity**			
Whites	0.22 (0.18, 0.26), < 0.001	0.09 (0.04, 0.13), < 0.001	0.07 (0.02, 0.11), 0.009
Blacks	0.42 (0.34, 0.51), < 0.001	0.29 (0.19, 0.38), < 0.001	0.18 (0.08, 0.27), < 0.001
Mexican Americans	0.41 (0.33, 0.49), < 0.001	0.26 (0.17, 0.34), < 0.001	0.25 (0.16, 0.35), < 0.001
Other race/ethnicity	0.36 (0.30, 0.42), < 0.001	0.20 (0.14, 0.27), < 0.001	0.15 (0.08, 0.22), < 0.001

Model 1: no covariates were adjusted. Model 2: Age, sex, and race/ethnicity were adjusted. Model 3: Age, sex, race/ethnicity, education level, income poverty ratio, BMI, waist circumference, physical activity, smoking behavior, alcohol behavior, alanine aminotransferase, aspartate aminotransferase, high-density lipoprotein cholesterol, total cholesterol, triglycerides, low-density lipoprotein cholesterol, total protein, hypertension, diabetes, and weak/failing kidneys were adjusted. In the subgroup analysis stratified by gender or race/ethnicity, the model was not adjusted for the stratification variable itself

In the subgroup analysis (Table 3), the association between sUA and CPK did not reach statistical significance in the female population except in other race/ethnicity subjects (*p* for trend < 0.001). In the male population, the sUA level was significantly associated with CPK in Black (*p* for trend < 0.001) and Mexican American subjects (*p* for trend < 0.001).

**Table 3:** Subgroup analysis stratified by race and sex

Quartiles of serum uric acid	Creatine phosphokinase IU/L (95% CI)			
	**Whites**	**Blacks**	**Mexican Americans**	**Other race/ethnicity**
**Male**				
Lowest quartiles	141.5(119.9,163.1)	229.8(182.0, 277.7)	91.8 (83.4, 100.3)	189.5 (160.8, 218.2)
2nd	142.2(129.2,155.1)	255.7(228.0, 283.5)	106.0 (96.2, 115.8)	171.4 (153.8, 189.1)
3nd	152.6(141.8,163.3)	251.2(229.0, 273.4)	109.4 (96.9, 121.8)	180.9 (165.1, 196.7)
4nd	153.9(143.6,164.2)	308.1(288.4, 327.8)	97.4 (74.2, 120.6)	192.0 (178.0, 205.9)
*p* for trend	0.130	< 0.001	< 0.001	0.249
**Female**				
Lowest quartiles	91.8 (86.3, 97.3)	153.5(135.9, 171.2)	91.9 (83.4, 100.4)	100.3 (92.6, 107.9)
2nd	96.2 (90.4, 101.9)	162.4(144.6, 180.2)	106.3 (96.4, 116.1)	113.5 (105.5, 121.4)
3nd	89.3 (81.9, 96.8)	164.7(143.9, 185.6)	108.8 (96.3, 121.3)	122.2 (111.7, 132.7)
4nd	87.9 (78.3, 97.5)	184.1(161.2, 207.1)	97.3 (73.7, 121.0)	125.4 (109.0, 141.8)
*p* for trend	0.467	0.072	0.061	< 0.001

Age, sex, race/ethnicity, education level, income poverty ratio, BMI, waist circumference, physical activity, smoking behavior, alcohol behavior, alanine aminotransferase, aspartate aminotransferase, high-density lipoprotein cholesterol, total cholesterol, triglycerides, low-density lipoprotein cholesterol, total protein, hypertension, diabetes, and weak/failing kidneys were adjusted

**Fig. 3:**
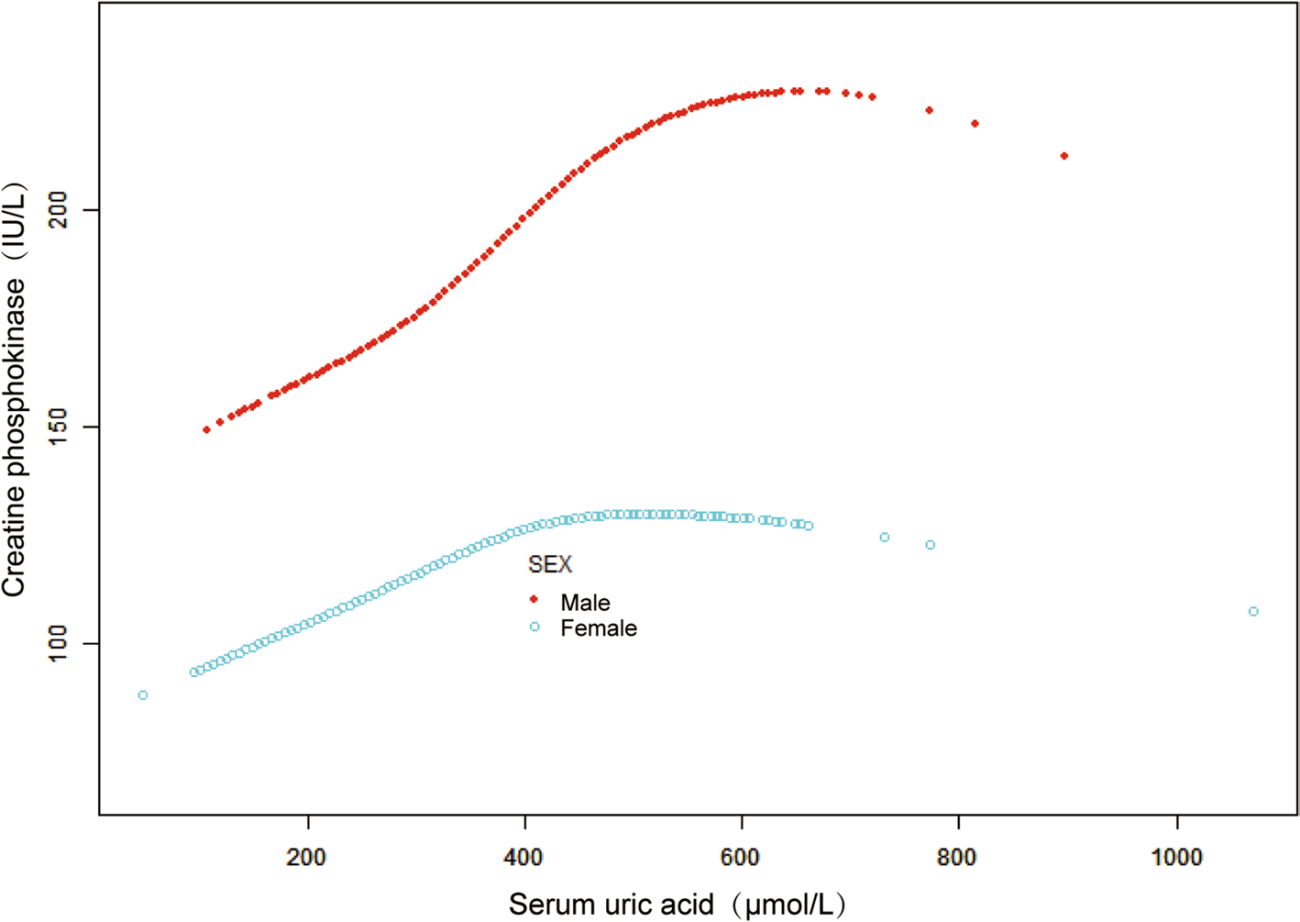
The relationship between serum uric acid level and creatine phosphokinase dose–response, stratified by age. Sex, race/ethnicity, education level, income poverty ratio, BMI, waist circumference, physical activity, smoking behavior, alcohol behavior, alanine aminotransferase, aspartate aminotransferase, high-density lipoprotein cholesterol, total cholesterol, triglycerides, low-density lipoprotein cholesterol, total protein, hypertension, diabetes, and weak/failing kidneys were adjusted. Significance of smooth terms: *p* < 0.001 (Male); *p* = 0.001 (Female)

**Fig. 4:**
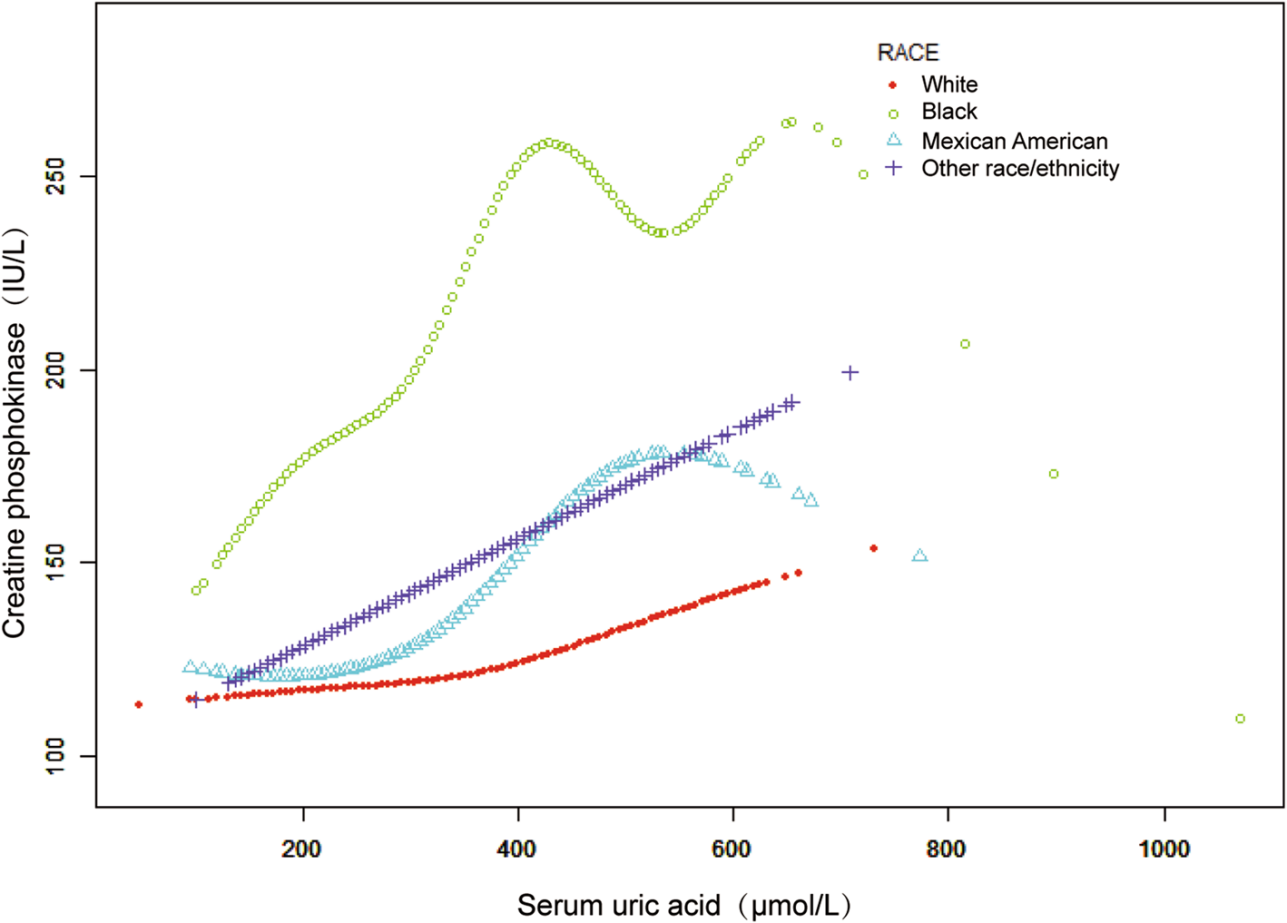
The relationship between serum uric acid level and creatine phosphokinase dose–response, stratified by race/ethnicity. Age, sex, education level, income poverty ratio, BMI, waist circumference, physical activity, smoking behavior, alcohol behavior, alanine aminotransferase, aspartate aminotransferase, high-density lipoprotein cholesterol, total cholesterol, triglycerides, low-density lipoprotein cholesterol, total protein, hypertension, diabetes, and weak/failing kidneys were adjusted. Significance of smooth terms: *p* = 0.123 (White); *p* < 0.001 (Black); *p* < 0.001 (Mexican American); *p* < 0.001 (Other race/ethnicity)

In addition, we used weighted additive models and smooth curve fitting to identify nonlinear relationships between sUA and CPK, stratified by sex and race/ethnicity (Figs. 3 and 4). The association between sUA and CPK followed an inverted U-shaped curve in females (*p* = 0.001) (turning point: sUA = 428.3 μmol/L) (Table 4, Fig. 3). The test for interaction was significant between the two sexes (*p* = 0.039) (Table 4). CPK increased with sUA levels in the male population, Black, Mexican American, and other race/ethnicity (*p* < 0.001 for all) (Figs. 3 and 4). However, smooth curve fitting of the nonlinear relationships between sUA and CPK did not reach statistical significance in the White (*p* = 0.123) (Fig. 4).

**Table 4:** Threshold effect analysis of serum uric acid on creatine phosphokinase using two-piecewise linear regression

Creatine phosphokinase (IU/L)	Adjusted ß (95% CI), *p* value
**Female**	
Serum uric acid < 428.3 μmol/L	0.10 (0.00, 0.10), 0.001
Serum uric acid > 428.3 μmol/L	-0.10 (-0.20, 0.00), 0.198
*p* interaction	0.039

Age, sex, race/ethnicity, education level, income poverty ratio, BMI, waist circumference, physical activity, smoking behavior, alcohol behavior, alanine aminotransferase, aspartate aminotransferase, high-density lipoprotein cholesterol, total cholesterol, triglycerides, low-density lipoprotein cholesterol, total protein, hypertension, diabetes, and weak/failing kidneys were adjusted. In the subgroup analysis for females and other races/ethnicities, the model was not adjusted for gender or race/ethnicity, respectively

## Discussion

In this study, we used a large nationally representative sample of the US population to investigate whether the sUA level is independently associated with CPK. The results showed that sUA was positively correlated with CPK after adjusting for potential confounders. sUA had a positive relationship with CPK in both male and female populations, White, Black, Mexican American, and other races/ethnicities. The association between sUA and CPK followed an inverted U-shaped curve in females (turning point: sUA = 428.3 μmol/L), and the sex interaction term was significant (*p* = 0.039).

Previous studies have found an association between sUA and abnormal electrocardiogram (ECG) signs. A Brisighella Heart Cohort Study found that sUA predicted incident tachyarrhythmias, Q waves, and electrocardiogram (ECG) signs of left ventricular hypertrophy (LVH) [21]. Liu et al. [22] demonstrated that the dose–response effects of sUA were associated with the prevalence of electrocardiographic LVH in healthy individuals. In addition, elevated sUA was associated with ischemic changes and arrhythmias in ECG in the general population [23]. In a 10-year follow-up, Cuspidi et al. [24] demonstrated that sUA was a predictor of long-term echocardiographic changes from normal left ventricular mass index to LVH. The QT interval in ECG mainly reflects cardiac ventricular repolarization. Prolonged heart rate-corrected QT interval has been shown as a marker of CVD death and sudden cardiac death [25]. Mozos et al. [26] found that sUA was associated with prolonged QT interval in patients with liver cirrhosis. High sUA levels may adversely affect depolarization and repolarization of the heart by causing subclinical endocardial myocardial changes [27].

The positive relationship between sUA and CPK we observed might be due to the triggering of myocardial cell damage by sUA to some extent. It has been found that sUA can activate NLRP3 inflammasome and induce oxidative stress [28–30]. The NLRP3 inflammasome is a ubiquitous intracellular pattern recognition receptor that is critically involved in the response to injury during AMI [31]. By activating NF‐κB and MAPK signaling pathways, sUA induced the release of proinflammatory cytokines, such as IL‐18 and IL‐1β, which are mainly regulated by the activation of NLRP3 inflammasome [32]. One study has shown that sUA could induce cardiomyocyte damage through activating NLRP3 inflammasome in a TLR6/NF‐κB/p65‐dependent manner [33]. Oxidative stress can cause an imbalance between free radical generation and elimination due to increased production of reactive oxygen and nitrogen species generation and/or insufficient antioxidant defense, thus leading to myocardial injury [34]. It is known that xanthine oxidase activity is enhanced in patients with hyperuricemia. The production of free radicals caused by xanthine oxidase is responsible for reperfusion arrhythmia [35]. As the most common antioxidant in serum, sUA plays a role in inflammation and atherogenesis, which affects the pathogenesis of AMI [36]. A cohort study reported that oxidative stress induced by hyperuricemia may lead to reperfusion arrhythmias in acute MI [37]. Increasing evidence supports that sUA can increase the sensitivity of myocardial cells to myocardial ischemia–reperfusion (MI/R) injury [38]. It has been reported that sUA aggravates MI/R injury through ROS/NLRP3 focal bypass [39].

An interesting finding was that the relationship between sUA levels and CPK in females followed an inverted U-shaped curve. For females, CPK increases with sUA until the turning point (sUA = 428.3 μmol/L). It was observed that there was a different relationship between sUA and CVD in different genders. A Framingham Heart Study showed that elevated sUA level was not associated with an increased risk of adverse outcomes in men, while sUA level was a predictor of coronary heart disease and death from CVD in women [40]. Kawabe et al. [41] found that increased UA levels in women were more strongly associated with major cardiovascular adverse events of acute coronary syndrome than in men. Studies demonstrated that high sUA was an independent predictor of all-cause mortality in women with chronic heart failure but not in men [42]. However, Guo et al. [27] proposed that high sUA were prone to a higher risk for prolonged heart rate-corrected QT intervals, but not in the female group. It has been shown that men have higher levels of sUA circulation than women [43]. The lower levels of sUA in women were caused by the increased fraction of uric acid excretion induced by estrogen [44]. This sex difference may lead to different relationships in CPK with the increase of sUA level. The inverted U-shaped curve between sUA and CPK found in women in this study may be due to differences in gender, study sample, ethnic distribution, and confounding variables controlled.

To our knowledge, this study is the first to describe an association between sUA and CPK levels, revealing a possible association in acute myocardial cell injury. The strengths of this study are the population-based design, the large sample size, and the extensive confounding factors. In this study, we analyzed a representative sample of the multiracial population to better generalize the US population and provide reliable results. In addition, when exploring the relationship between sUA and CPK, we fully adjusted for potential confounding factors and conducted a subgroup analysis stratified by race and sex. However, our study also has some limitations. First, due to limited data, we could only study the correlation between sUA and CPK at a single point in time, rather than longitudinal data. The cross-sectional nature of these findings does not provide causal inferences. Second, sUA measurements were only performed at one point in time and may be influenced by individual behavior at that time, especially high purine dietary consumption. Third, there is the possibility of bias caused by other potential confounding factors that we did not adjust for. We only excluded subjects with rhabdomyolysis (CPK > 1000 U/L). We did not indicate whether patients had additional diseases, such as diagnosed CVD, stroke, respiratory problems, cancer/malignancy, arthritis, and liver conditions.

In conclusion, our study suggests that sUA is positively correlated with CPK in the general population of the US. However, for the female population in the US, reaching a certain sUA level (turning point, 428.3 μmol/L) will not increase CPK. The association we found in this study is biologically plausible, and fundamental research and longitudinal studies with large sample sizes are needed to confirm the mechanism and impact of sUA levels on the prediction of CPK elevation.

## Data Availability

The survey data are publicly available on the internet for data users and researchers throughout the world(www.cdc.gov/nchs/nhanes/).

## Abbreviations

sUA: Serum uric acid
CPK: Creatine phosphokinase
AMI: Myocardial infarction
CVD: Cardiovascular disease
BMI: Body mass index
AST: Aspartate aminotransferase
ALT: Alanine aminotransferase
HDL-C: High-density lipoprotein cholesterol
TC: Total cholesterol
TG: Triglycerides
LDL-C: Low-density lipoprotein cholesterol
ECG: Electrocardiogram
LVH: Left ventricular hypertrophy
MI/R: Myocardial ischemia–reperfusion
